# Establishment of single-cell transcriptional states during seed germination

**DOI:** 10.1038/s41477-024-01771-3

**Published:** 2024-09-10

**Authors:** Lim Chee Liew, Yue You, Lucas Auroux, Marina Oliva, Marta Peirats-Llobet, Sophia Ng, Muluneh Tamiru-Oli, Oliver Berkowitz, Uyen Vu Thuy Hong, Asha Haslem, Tim Stuart, Matthew E. Ritchie, George W. Bassel, Ryan Lister, James Whelan, Quentin Gouil, Mathew G. Lewsey

**Affiliations:** 1 La Trobe Institute for Sustainable Agriculture and Food, AgriBio, La Trobe University, Melbourne, Victoria Australia; 2 Epigenetics and Development Division, The Walter and Eliza Hall Institute of Medical Research, Melbourne, Victoria Australia; 3 Department of Medical Biology, The University of Melbourne, Melbourne, Victoria Australia; 4 Australian Research Council Centre of Excellence in Plant Energy Biology, School of Molecular Sciences, The University of Western Australia, Perth, Western Australia Australia; 5 Australian Research Council Centre of Excellence in Plants for Space, School of Molecular Sciences, The University of Western Australia, Perth, Western Australia Australia; 6 Australian Research Council Research Hub for Medicinal Agriculture, AgriBio, La Trobe University, Melbourne, Victoria Australia; 7 Genome Institute of Singapore, Agency for Science, Technology and Research (A*STAR), Singapore, Republic of Singapore; 8 School of Life Sciences, University of Warwick, Coventry, UK; 9 Harry Perkins Institute of Medical Research, Queen Elizabeth II Medical Centre and Centre for Medical Research, The University of Western Australia, Perth, Western Australia Australia; 10 Australian Research Council Centre of Excellence in Plant Energy Biology, AgriBio Building, La Trobe University, Melbourne, Victoria Australia; 11 College of Life Sciences, Zhejiang University, Hangzhou, People’s Republic of China; 12 Australian Research Council Centre of Excellence in Plants for Space, AgriBio, La Trobe University, Melbourne, Victoria Australia

**Keywords:** Biological techniques, Plant molecular biology

## Abstract

Germination involves highly dynamic transcriptional programs as the cells of seeds reactivate and express the functions necessary for establishment in the environment. Individual cell types have distinct roles within the embryo, so must therefore have cell type-specific gene expression and gene regulatory networks. We can better understand how the functions of different cell types are established and contribute to the embryo by determining how cell type-specific transcription begins and changes through germination. Here we describe a temporal analysis of the germinating *Arabidopsis thaliana* embryo at single-cell resolution. We define the highly dynamic cell type-specific patterns of gene expression and how these relate to changing cellular function as germination progresses. Underlying these are unique gene regulatory networks and transcription factor activity. We unexpectedly discover that most embryo cells transition through the same initial transcriptional state early in germination, even though cell identity has already been established during embryogenesis. Cells later transition to cell type-specific gene expression patterns. Furthermore, our analyses support previous findings that the earliest events leading to the induction of seed germination take place in the vasculature. Overall, our study constitutes a general framework with which to characterize *Arabidopsis* cell transcriptional states through seed germination, allowing investigation of different genotypes and other plant species whose seed strategies may differ.

## Abstract (web summary)

This study reports a transcriptome atlas of the germinating *Arabidopsis* embryo at single-cell resolution. It demonstrates that the cells of the embryo transition from a common transcriptional state to cell type-specific states as germination progresses.

## Main

Germination is the process through which seeds begin to grow and establish in their environment, which is fundamental to agricultural production. Success requires that the seed monitors both its internal resources and the surrounding, external conditions to co-ordinate appropriate responses based on this information and ensure that the time and place are suitable for future plant growth. Germination and early seedling growth are consequently plastic, dynamic processes.

The seed is a complex structure comprising many tissues and cell types, which are established during embryogenesis^1–3^. These cell types have individual functions and biochemistry that enable tight spatiotemporal control of growth and development. Variation in temperature is sensed and interpreted as a signal to germinate by just tens of cells in the embryonic radicle of dormant *Arabidopsis* seeds, in conjunction with the monolayer of cells in the endosperm^4,5^. Growth is then driven by cell expansion once germination commences, with both spatial and temporal variation in expansion rates. Initially, growth occurs in cells adjacent to the radicle tip, then proceeds to include cells further along the radicle and in the hypocotyl^6^. The end of germination is defined by emergence of the radicle through the endosperm and testa (seed coat), following which the cotyledons emerge and the seedling transitions from heterotrophic to photoautotrophic growth^7^.

Spatiotemporal control of growth and development requires correspondingly precise regulation of gene expression. The nuclei of mature *Arabidopsis* embryo cells condense and transition to a heterochromatic state by the end of seed development, repressing gene expression^8–10^. This is thought to be an adaptation enabling seeds to tolerate the desiccation that occurs at the end of seed development^9^. The nuclei then reverse this process as the seed imbibes water and germination commences, decondensing and transitioning to the euchromatic state required for gene transcription^9,11^. However, germination considered in the strict sense (that is, to the point of radicle protrusion) is thought to be dependent only on translation, whereas de novo transcription during germination is non-essential^12^. Consistent with this, mature *Arabidopsis* seeds contain populations of stored transcripts that were transcribed during seed development, a subset of which are translated early in germination^12–14^. During this time, the developing embryo draws on stored energy reserves, primarily in the form of lipids, but also from cell wall carbohydrates^8,15–18^. Nonetheless, transcription and de novo gene expression occur relatively early in germination, within 1–2 h of imbibition^19,20^. A cascade of transcription factors regulate gene expression at this time, which influences the rate at which germination progresses and is essential for a successful transition to post-germination seedling establishment^5,13,19,21,22^. Temporal changes occur in DNA methylation and small RNA abundance during germination, both of which are likely to be involved in gene regulation^13,23,24^. Gene expression also varies spatially within seeds, reflecting the different functions of tissues and cell types^1,4,25–27^. However, the resolution of spatial studies, and consequently the insight they provide, have been limited by the precision and scale achieved by the predominant methods of hand isolation or laser capture microdissection^1,25,26^.

Through understanding the spatiotemporal regulation of gene expression in seeds, we can better understand how the functions of different cell types are specified, and how these contribute to the functions of the seed as a whole. To this end, we investigated gene expression dynamics in the *Arabidopsis* embryo over the first 48 h of germination, as it transitions into a seedling, at single-cell resolution. We then interrogated the data to understand how the transcriptional state of cells progresses and how gene expression is regulated. We observed that most cells pass through a shared early transcriptional state before transitioning to cell type-specific transcriptional states. Once established, these cell type-specific transcriptional states were dynamic over germination and reflected changing functional properties of the cells. We constructed gene regulatory models for each cell type, from which we predicted key transcription factors that are active in individual cell types. This study provides insight into the different regulatory mechanisms operating as a seedling establishes itself within the environment.

## Results

### Generation of a single-cell expression atlas of germinating embryos

**Fig. 1:**
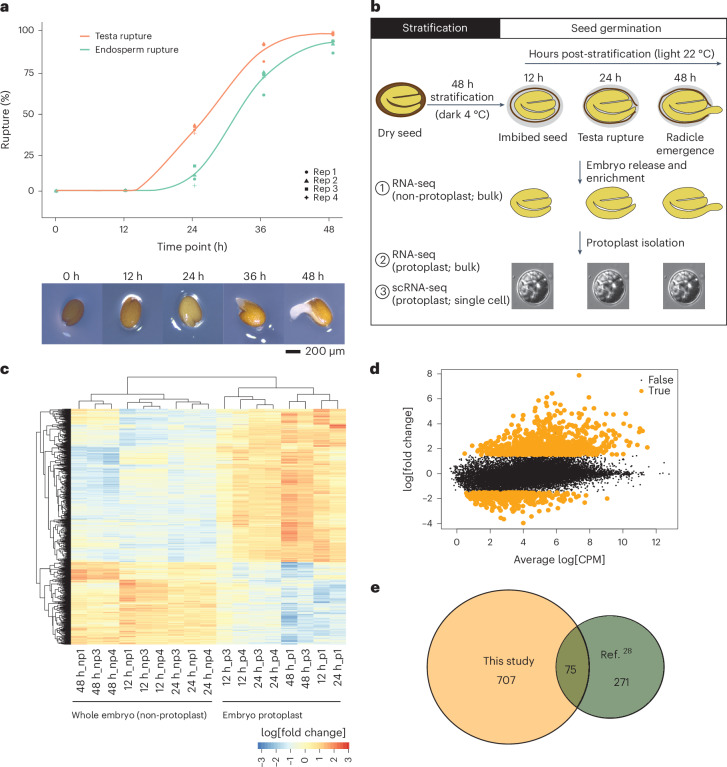
Germinating embryo scRNA-seq experimental design and impact of protoplast isolation on the transcriptome. **a**, *Arabidopsis* seed germination analysis showing the percentage rupture of different stages during seed germination by measuring testa rupture (red line) and endosperm rupture (green line). Representative images for each time point are shown below. **b**, Germination procedure and sampling for transcriptomic analyses: (1) RNA-seq of whole isolated embryos without protoplast isolation; (2) RNA-seq of embryo protoplasts; and (3) scRNA-seq of individual embryo protoplasts. **c**,**d**, Expression changes of 1,202 genes in response to protoplast isolation (log_2_[fold change]). The transcriptional response to protoplast isolation was consistent across time points (heatmap (**c**)) and included large fold-changes for well expressed genes (MA-plot (**d**)). **e**, Venn diagram of the limited overlap in upregulated genes upon protoplast isolation between this study and a previous study by Birnbaum and colleagues^28^. CPM, counts per million; np, non-protoplast; p, protoplast. Source data

The major goals of our study were to characterize how gene expression differs between the cell types that constitute germinating *Arabidopsis* embryos and to determine how these patterns of gene expression may be regulated. To this end, we generated a single-cell RNA sequencing (scRNA-seq) atlas of germinating *Arabidopsis* embryos (Fig. 1). First, we established baseline germination dynamics in our system by performing a germination assay with Columbia-0 (Col-0) seeds, assessing testa and endosperm rupture over 48 h after transfer to light (Fig. 1a). Neither testa rupture nor endosperm rupture was observed at 12 h; at 24 h, there was 41% testa rupture and 12% endosperm rupture, followed at 36 h by 89% testa rupture and 71% endosperm rupture and at 48 h by 96% testa rupture and 92% endosperm rupture. We selected three time points for analysis from this assay (12, 24 and 48 h), corresponding to early germination (imbibed seed), mid-germination (the start of testa rupture) and the end of germination (endosperm rupture and radicle emergence) (Fig. 1a,b)^13^. Seeds were harvested at each time point and embryos were released from the seed coat and endosperm by physical disruption. Protoplasts of enriched embryo cells were then isolated and enriched to high purity to physically separate the cells from one another and make them amenable to microfluidic handling.

Isolation of protoplasts affects gene expression in sampled cells, creating a technical effect that must be controlled for in subsequent analyses^28–30^. To enable this correction, we analysed the effect of protoplast isolation on embryo transcriptomes using bulk RNA-seq (that is, not scRNA-seq). We compared transcriptomes in whole, isolated embryos with those of our protoplast preparation, finding that 1,202 genes were differentially expressed between them (1% false discovery rate (FDR); log_2_[fold change] > 1.5; Fig. 1c,d and Supplementary Table 1). The genes responsive to protoplast isolation responded consistently across all time points (Fig. 1c) and the overall transcription dynamics during germination were preserved in protoplasts (only 0.4–2.5% of discordant changes between time points, where a gene that is upregulated in the whole-embryo data is downregulated in the protoplast data, or vice versa). Removing protoplast-responsive genes slightly improved the correlation between whole-embryo transcriptomes and single-cell pseudobulk transcriptomes at each time point (Pearson correlation coefficients of 0.80, 0.83 and 0.80 for the 12, 24 and 48 h time points, respectively, up from 0.79, 0.82 and 0.79) (Supplementary Table 2). These genes were excluded from our subsequent scRNA-seq data analyses.

Other plant single-cell gene expression studies have also used protoplast isolation to separate cells^28–38^. Approaches to correct for the effect of protoplast isolation have varied. Some studies have applied bulk RNA-seq in the same manner as ourselves^29,30,34,35,37,38^, whereas others have either used a single previously generated reference set of protoplast isolation-responsive genes^28,31,36^ or reported no correction at all^32,33^. Other scRNA-seq studies that have been comparably rigorous have detected similar numbers of protoplast-responsive genes (2,231 genes^38^ and 3,545 genes^29^), compared with the 1,202 genes that we detected in our study. It is possible that the population of protoplast isolation-responsive genes varies depending on experimental conditions, growth stage and other factors. We tested this by determining how many of the responsive genes were shared between our analysis and the previously generated reference set^28^. Only 75 of our 782 upregulated protoplast isolation-responsive genes were shared between the two datasets (Fig. 1e). This indicates that systematic parallel analysis and control for the effects of cell isolation is important when conducting scRNA-seq experiments.

**Fig. 2:**
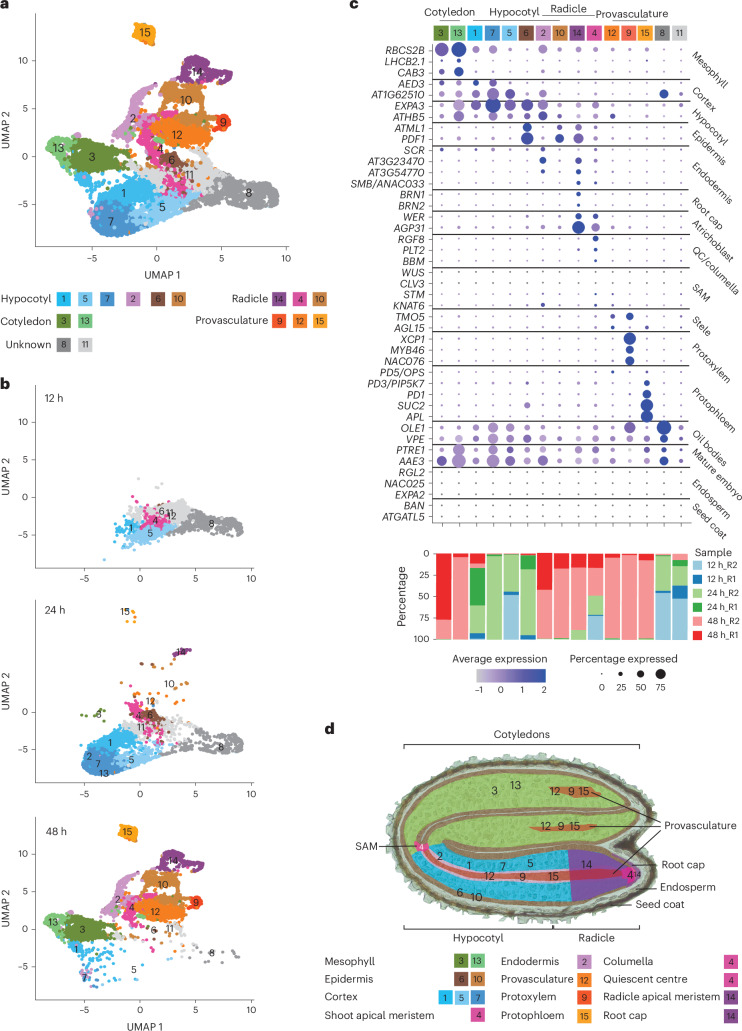
Annotation of germinating embryo cell types using literature-curated marker transcripts. **a**, Uniform manifold approximation and projection (UMAP) dimensional reduction and visualization of 12,798 cells in 15 clusters. **b**, UMAP dimensional reduction and visualization of cells across three time points—12, 24 and 48 h after placement to light—showing the temporal changes in cell and cluster detection. **c**, Bubble plots showing the enrichment of expression of representative cell type-specific marker transcripts in 15 clusters and the percentage of cells from the two biological replicates (replicate 1, R1; replicate 2, R2) within each cluster at each time point. Marker transcripts were identified from published studies. **d**, Spatial distribution of cell clusters in the *Arabidopsis* embryo. SAM, shoot apical meristem; QC, quiescent centre. Source data

**Extended Data Fig. 1:**
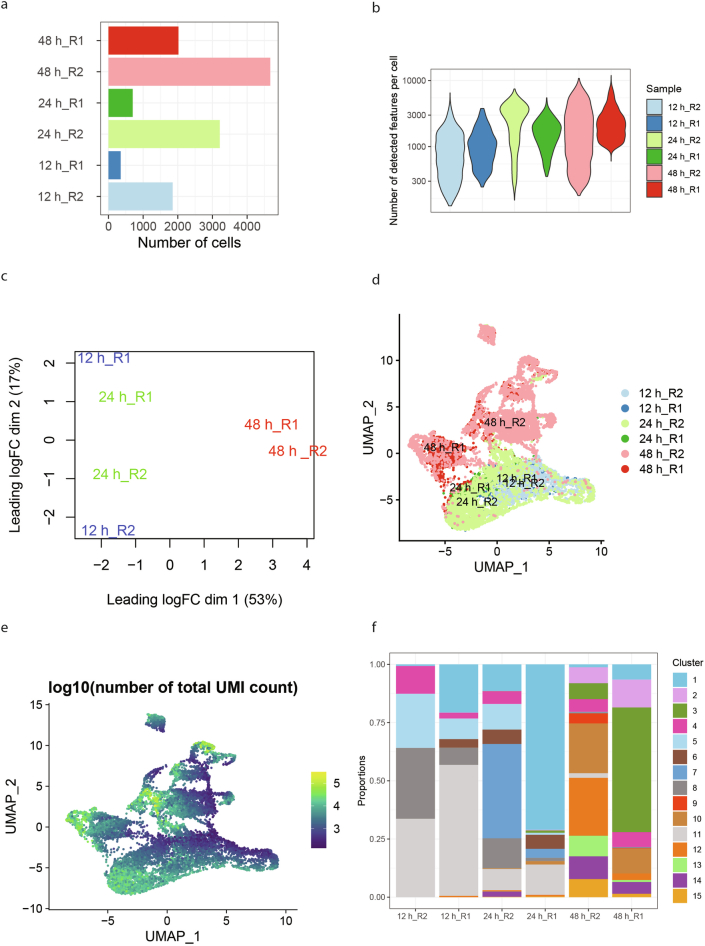
Quality assessment and integration of scRNA-seq data. **a**, Numbers of cells captured for each time point (12, 24, 48 h) and biological replicate (R1, R2) in Col-0. **b**, Distribution of number of detected genes per cell. **c**, MDS plot of pseudo-bulks for each scRNA-seq sample. **d**, UMAP plot depicting relative similarity of all cells post batch correction and data integration. **e**, Total Unique Molecular Identifier (UMI) count per cell for all cells. **f**, Proportional distribution of cells between clusters in each sample.

Transcriptomes of germinating embryo protoplasts were analysed using the 10X Genomics Chromium platform to produce a single-cell atlas (Fig. 2 and Extended Data Fig. 1). Two biologically independent replicates were conducted per time point, with each sample a pool of hundreds of embryos. Samples in the first replicate comprised an equal mixture of Col-0 and Cape Verde Islands-0 (Cvi-0) cells for technical reasons, to enable real single cells to be distinguished from doublet cell capture events and background noise. Real single cells would contain only single nucleotide polymorphisms (SNPs) from a single ecotype, whereas doublets and background would contain a mixture of SNPs from both ecotypes. This was particularly helpful for the early time points, where cell messenger RNA (mRNA) content was low and default settings of the 10X Genomics data processing pipelines may have missed transcriptionally quiescent cells. Only Col-0 cells were used for all subsequent biological analyses, as the germination properties of Cvi-0 differ substantially. This yielded a total of 12,798 Col-0 cells, with an average 1,025 expressed genes per cell (Extended Data Fig. 1a,b). Cell recovery and the number of detected genes increased with time of germination, although equal numbers of cells were loaded per sample and each sample was sequenced at a similar depth (Extended Data Fig. 1a; 140–170 million reads per sample). It is likely that this occurred because the number of transcripts per cell was low in the early stages of germination (fewer unique molecular identifiers (UMIs) detected despite higher numbers of reads per cell; Extended Data Fig. 1e), affecting the detection of true cells from background, even after fine-tuning the cell detection parameters using the two ecotypes. We integrated data from all samples to minimize technical effects, then clustered cells according to their transcriptional profiles and visualized the resulting clusters in two dimensions with uniform manifold approximation and projection to assess the consistency between replicates and time points (Fig. 2a and Extended Data Fig. 1c–e). Cells from independent replicates of individual time points were co-located in the analysis, indicating that replicates were consistent with one another after data integration (Extended Data Fig. 1d). Fifteen distinct clusters of cells with similar transcriptional profiles were resolved among these data, which probably represented different cell types and states (Fig. 2a).

The cell clusters detected within embryos differed markedly between the 12, 24 and 48 h time points (Fig. 2b and Extended Data Fig. 1f). No cell division occurs in embryos during germination, so no new cells arose in the time period studied^6^. Consequently, the differences in cell clusters between time points indicate that the transcriptional states of individual cell types within the embryo change over time during germination. Additionally, cell types are established during seed development rather than during germination. This means that the cell clusters defined in our experiment correspond to cell states adopted by the various cells and tissues through the time course of germination, rather than to cell types directly. The consistency between replicates at individual time points was considered (Extended Data Fig. 1d,f). Largely, the same clusters were present in each replicate, but the proportions of cells in those clusters varied. As gene expression is highly dynamic at this time, even a very small shift in harvest time or environmental conditions might cause differences between replicates. Overall, however, the replicates corresponded well with one another.

### Annotation of cell types in the expression atlas

We annotated the clusters within the embryo single-cell atlas so that we could interpret the changes occurring among them during germination (Fig. 2c and Supplementary Table 3). Annotation of single-cell data is often achieved by reference to an existing ground truth dataset of manually dissected or sorted cells from the organ of interest^28,31–33^. A comprehensive ground truth dataset does not exist that describes all cell types of the embryo during germination. To overcome this, we investigated the published literature and identified relevant marker genes from several studies, then compared them with genes expressed specifically in one or a small number of clusters (Supplementary Table 3).

We were able to infer identities for 13 of the 15 clusters (Fig. 2c,d). We detected the most abundant cell types of the cotyledon (mesophyll (clusters 3 and 13)), hypocotyl (cortex (clusters 1, 5 and 7), epidermis (cluster 6) and cortex/endodermis (cluster 2)) and radicle (epidermis (cluster 10), radicle apical meristem (cluster 14) and quiescent centre/columella (cluster 4)). Cell type markers were expressed clearly by cluster 2 (cortex/endodermis) and cluster 10 (epidermis), but we could not distinguish whether these were resident in the hypocotyl or radicle, probably reflecting that these cell types are continuous between the two organs at this developmental stage. Cells of the provasculature (protophloem (cluster 15), protoxylem (cluster 9) and provascular cells (cluster 12)) were mostly detected at the 48 h time point. Identities could not be assigned to clusters 8 and 11 because they did not show clear enrichment of expression for any marker genes from the literature, indicating that the clusters may represent some uncharacterized cell state or type. Clusters strongly expressing the marker transcripts of the shoot apical meristem *WUSCHEL* (*WUS*; AT2G179500), *CLAVATA3* (*CLV3*; AT2G27250), *SHOOT MERISTEMLESS* (*STM*; AT1G62360) and *KNOTTED1-LIKE HOMEOBOX GENE 6* (*KNAT6*; AT1G23380) were not detectable in the dataset. Some enrichment of *KNAT6* and *STM* was detected in clusters 2, 4 and 12, suggesting that these clusters might include a small number of shoot apical meristem cells. The absence of a clear shoot apical meristem cluster probably occurred because these cells are very rare (approximately eight cells) relative to the total number of cells in an embryo. We also did not detect expression of endosperm or seed coat marker in any cluster, confirming the successful isolation and enrichment of embryos (Fig. 2c).

**Fig. 3:**
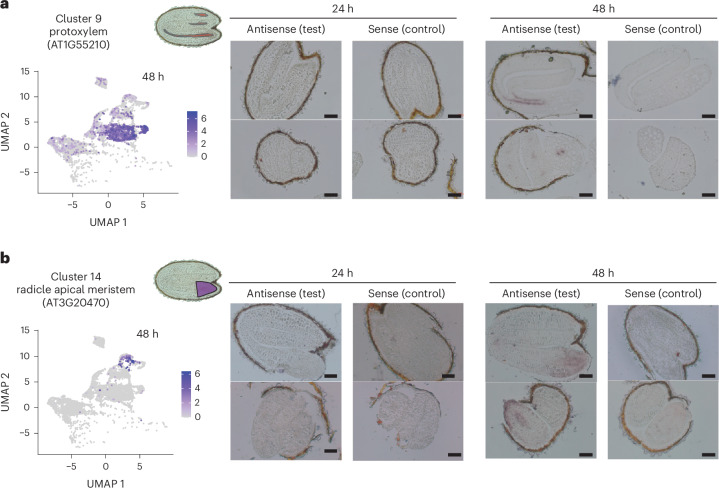
Validation of cell type annotation using RNA in situ hybridization. **a**,**b**, Expression domains of independent marker transcripts of cluster 9 (**a**) and cluster 14 (**b**) confirmed the physical location of the cells in these clusters. UMAP dimensional reduction graph visualized the expression of marker transcript at 48 h. The hybridization signals were detected at 48 but not 24 h, also confirming the temporal detection of these clusters in scRNA-seq data. For each time point, the results of hybridization with antisense probes (that is, the test) and sense probes (negative controls) are shown. Representative images are shown (more than ten embryos were examined). Scale bars, 200 μm.

**Extended Data Fig. 2:**
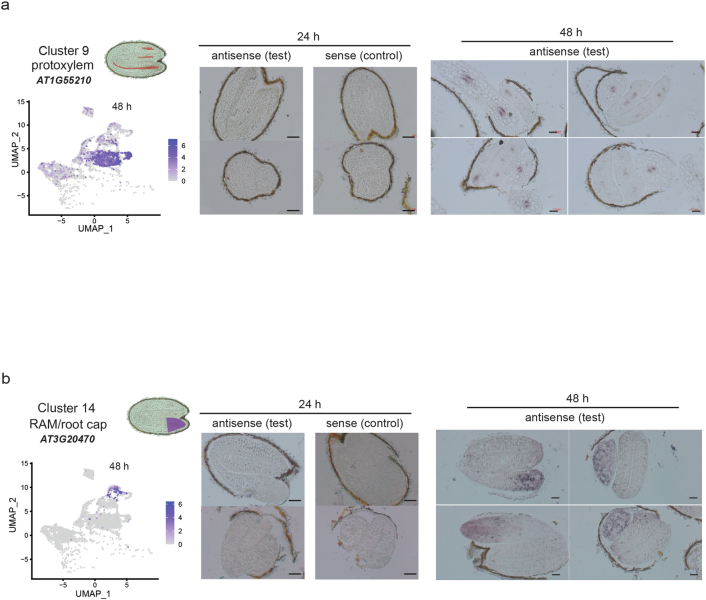
RNA in situ hybridisation of marker transcripts specific to cluster 9 (**a**) and cluster 14 (**b**). Representative images are shown with more than 10 embryos examined. Scale bars indicate 200 μm.

We validated annotations for clusters 9 and 14 using an independent experimental method (Fig. 3). We had annotated cluster 9 as protoxylem within the provasculature and present only at 48 h. The scRNA-seq analysis indicated that AT1G55210 expression was an independent marker for cluster 9, which had not been used in the initial literature-based annotation of the cluster, so we determined the location of its transcripts by RNA in situ hybridization (Fig. 3a, Supplementary Tables 4 and 5 and Extended Data Fig. 2). Correspondingly, a signal was detected specifically within the protoxylem at 48 h but was not detected at 24 h. We also validated the annotation of cluster 14 as radicle apical meristem cells, present only at 48 h. The location of the independent marker transcript AT3G20470 was examined. A signal was specifically detected in cells of the radicle apical meristem region and only at 48 h, but with the signal weaker in the radicle cortex cells (Fig. 3b, Supplementary Tables 4 and 5 and Extended Data Fig. 2). This observation corresponded with our annotation of cluster 14 from known marker transcripts, which indicated the presence of epidermis, endodermis, atrichoblast and root cap marker transcripts (Fig. 2c). The marker gene validation results for both clusters were also consistent with the changes in the detection of clusters over time described above, further illustrating the dynamic nature of cell transcriptomes during germination.

### Embryo cells undergo extensive transcriptional reprogramming

**Fig. 4:**
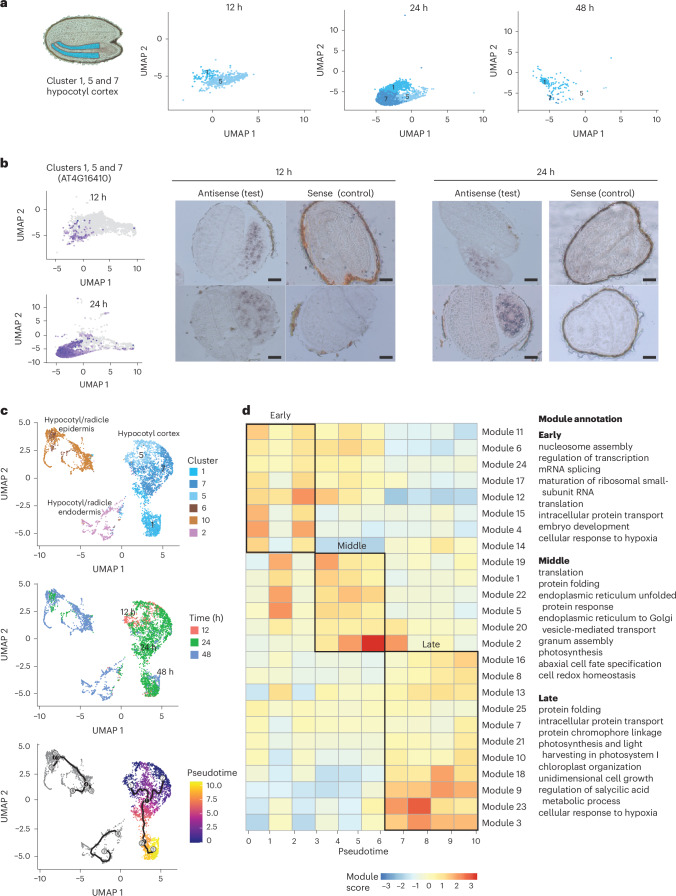
Clusters 1, 5 and 7 define a trajectory of hypocotyl cortex cell states. **a**, UMAP dimensional reduction and visualization of clusters 1, 5 and 7 cells across three timepoints, 12 h, 24 h and 48 h. **b**, Confirmation of the locations of clusters 1, 5 and 7 using RNA in situ hybridization of a marker transcript specific to these clusters at 12 and 24 h. Left, UMAP dimensional reduction plots of the expression of the marker transcript of AT4G16410 across all cells at each time point. Right, locations of the hypocotyl cortex cell type. Representative images are shown (more than ten embryos were examined). Scale bars, 200 μm. **c**, Cells of clusters 1, 5 and 7 form a contiguous group together. They sit upon a temporal trajectory from 12 h to 24 h to 48 h, which corresponds to the transition from cluster 5 cells, through cluster 7 to cluster 1 cells (top graph). Reconstruction of pseudotime (bottom graph) follows a trajectory that corresponds to the real time of germination (middle graph). On the trajectory, branch points are denoted by black circles and leaves (outcome cell states) are denoted by grey circles, while numbers are for reference purposes only. **d**, Co-expressed gene modules across the pseudotime trajectory of cluster 1, 5 and 7 cells. Early pseudotime is equivalent to early germination. The module annotations are major representative Gene Ontology terms associated with the modules, assessed using Gene Ontology term reduction. Source data

**Extended Data Fig. 3:**
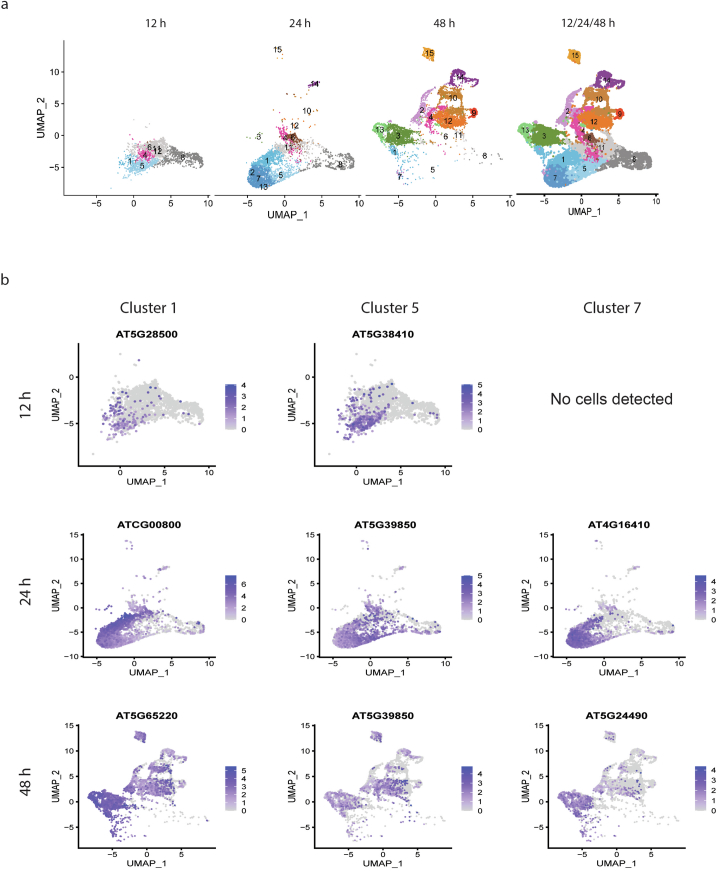
Identification of marker transcripts for clusters 1, 5 and 7. **a**, UMAP dimensional reduction and visualisation of cells at three individual time points, 12 h, 24 h, 48 h, and all time points together. **b**, Most highly specific marker transcripts for each of clusters 1, 5 and 7 individually. The plots illustrate that marker transcripts highly specific to each of these clusters individually could not be identified, likely due to high similarity in transcriptomes between the three clusters. These most highly individual cluster-specific marker transcripts were still expressed in clusters other than 1, 5 and 7. Source data

**Extended Data Fig. 4:**
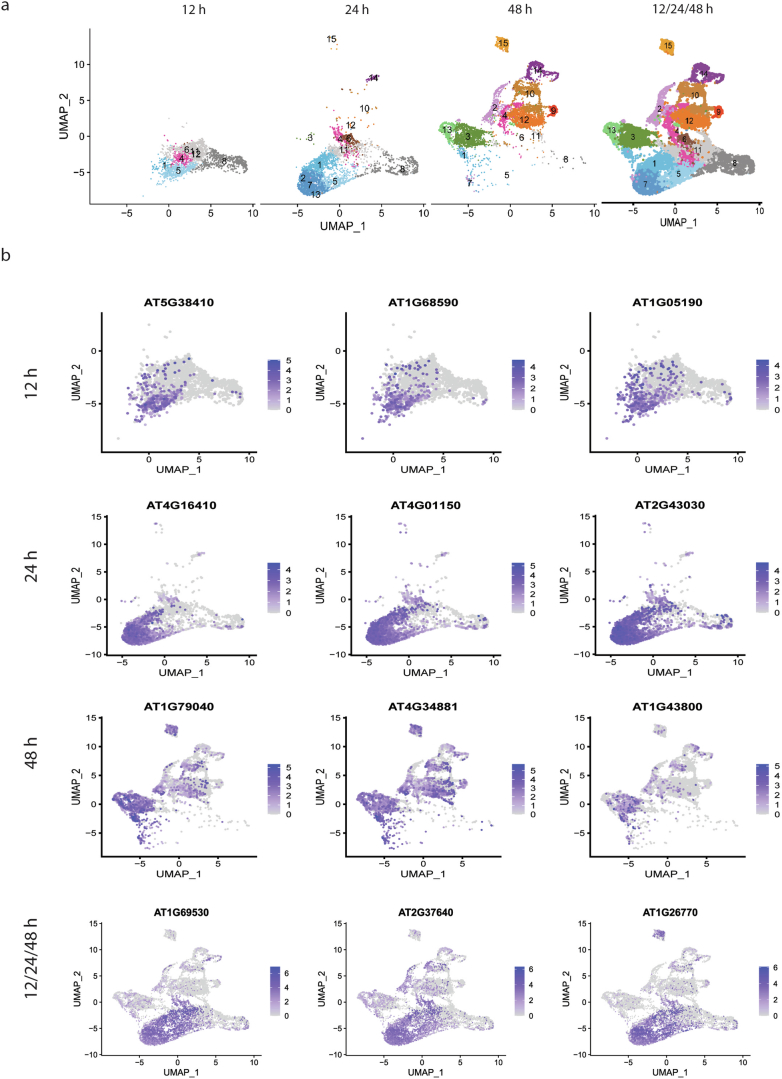
Identification of combined marker transcripts for clusters 1, 5 and 7. **a**, UMAP dimensional reduction and visualisation of cells at three individual time points, 12 h, 24 h, 48 h, and at all time points together. **b**, Most highly specific marker transcripts for clusters 1, 5, 7 combined. Marker transcripts identified were more highly specific to the clusters when these clusters were analysed as a group. Source data

**Extended Data Fig. 5:**
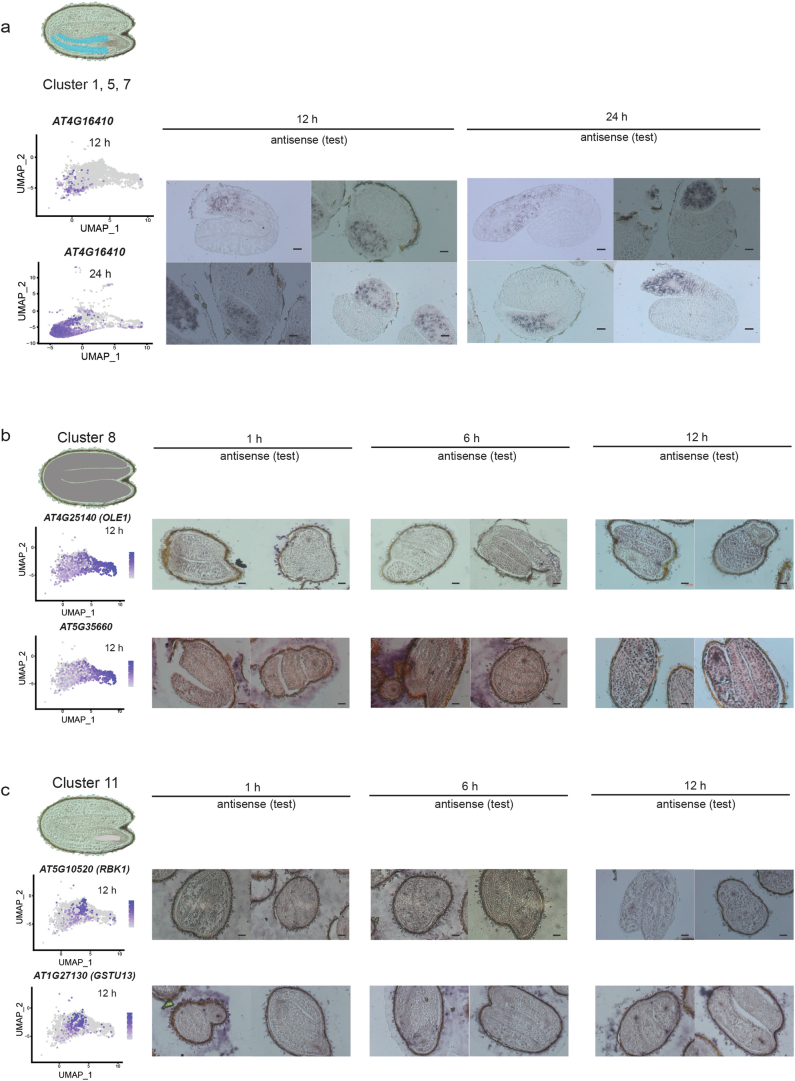
RNA in situ hybridisation of marker transcripts. **a**–**c**, Results of RNA in situ hybridisation of marker transcripts specific to clusters 1, 5 and 7 combined (**a**), cluster 8 (**b**) and cluster 11 (**c**). The icon on the top summarizes the location of the cell type where the marker transcripts expressed. UMAP dimensional reduction plot of expression of the marker transcript across all cells at the corresponding time point. Representative images are shown with more than 10 embryos examined. Scale bars indicate 200 *μ*m.

The growth properties and development of individual cells within the embryo change as germination progresses^5,6^. We investigated how underlying dynamic gene expression might contribute to changes in the functional properties of cells during this time and how this relates to the transcriptional states observed in our data. We focused on the hypocotyl cortex cells because these were detected as three distinct clusters (1, 5 and 7) present in different proportions at each time point across germination (Fig. 4). Cells of cluster 5 were the most abundant at 12 h, accompanied by a small number of cluster 1 cells (Fig. 4a). Cells of clusters 1 and 7 were most abundant at 24 h, whereas at 48 h very few cells were detected from any of the clusters, but cells of cluster 1 were the most abundant (Fig. 4a). The existence of three distinct clusters of hypocotyl cortex cells indicated that there were populations of hypocotyl cortex cells whose transcriptomes differed. It was not possible to identify single marker transcripts clearly specific to individual clusters, suggesting that the differences between clusters were relatively subtle and related to quantitative differences in the expression of many genes rather than the complete presence or absence of certain transcripts (Extended Data Fig. 3 and Supplementary Table 6). However, marker transcripts strongly specific to all three of clusters 1, 5 and 7 combined were readily identified (Extended Data Fig. 4 and Supplementary Table 7). AT4G16410 was a specific marker transcript of clusters 1, 5 and 7 at the 12 and 24 h time points in our scRNA-seq data. Expression of the transcript was detected by RNA in situ hybridization in lower and middle hypocotyl cortex cells at 12 and 24 h, confirming the cell type annotation of clusters 1, 5 and 7 (Fig. 4b and Extended Data Fig. 5).

We conducted a detailed analysis of the transcriptomes of cells within each of the three hypocotyl cortex clusters (1, 5 and 7) to understand their similarities and differences. We performed a new dimension reduction analysis only for cells annotated to hypocotyl clusters (1, 2, 5, 6, 7 and 10) to remove the influence of the large transcriptional differences from cells of other organs or tissues, thereby allowing us to focus on the smaller differences between hypocotyl cells (Fig. 4c). Cells of the hypocotyl cortex clusters again formed a contiguous group even at this focused scale, confirming that the transcriptomes of clusters 1, 5 and 7 were highly similar (Fig. 4c). The temporal ordering of the group was preserved, transitioning from cells harvested at 12 h (cluster 5), through 24 h (clusters 7 and 1) to 48 h (cluster 1; Fig. 4a). These observations suggested that clusters 1, 5 and 7 represent hypocotyl cortex cells transitioning through different transcriptional states over time during germination.

We further examined the proposal that clusters 1, 5 and 7 represent hypocotyl cortex cells at different stages of germination by assembling the hypocotyl cortex cells onto a pseudotime trajectory. The cells of a single cell type within an individual embryo become active at different times during germination, initiating a dynamic gene expression program^5,39^. Consequently, the cells of a single cell type are spread across a shared developmental trajectory of gene expression, with each cell in a slightly different expression state. Furthermore, the precise time at which germination begins under uniform conditions differs between genetically identical seeds^40^. Each sample in our experiment was a pool of hundreds of embryos. Therefore, many cells of each cell type would have been present in each of our samples, and these cells would have been distributed across a continuum of that cell type’s developmental trajectory. This experimental design is advantageous because it allows us to reconstruct the developmental trajectory of gene expression using a technique called pseudotime analysis. Pseudotime analysis arranges the cells of a single-cell experiment into order according to their expression states, and thereby developmental progression. In our case, early pseudotime should correspond to early germination and late pseudotime to late germination, and the cells are ordered on a continuum between these points. This ordering of cells enables examination of how gene expression changes dynamically during germination.

Reconstruction of the pseudotime trajectory supported the notion that clusters 1, 5 and 7 represent hypocotyl cortex cells transitioning through different stages of germination, with pseudotime following a similar path to the true time of germination (Fig. 4c). Furthermore, the dynamics observed in transcriptomes across hypocotyl cortex cell clusters indicated that the functional properties of these cells change during germination. To examine this, we identified modules of genes that were co-expressed and assessed their functions and timing of expression. There were 25 distinct modules of co-expressed genes across the pseudotime trajectory of clusters 1, 5 and 7 (Fig. 4d and Supplementary Table 8). These were broadly categorized as early (modules 4, 6, 11, 12, 14, 15, 17 and 24), mid- (1, 2, 5, 19, 20 and 22) and late (3, 7, 8, 9, 10, 13, 16, 18, 21, 23 and 25) on the pseudotime trajectory, which can be considered as equivalent to early, mid- and late germination. Genes co-expressed during early germination were enriched for functions related to chromatin accessibility, transcription, RNA splicing and translation; during mid-germination, functions related to translation, protein maturation and photosynthesis were more strongly evident; and in late germination, photosynthesis was the dominant function. These analyses indicate that dynamic gene expression drives functional changes in hypocotyl cortex cells across germination.

### An initial cell transcriptional state is established early in germination

**Fig. 5:**
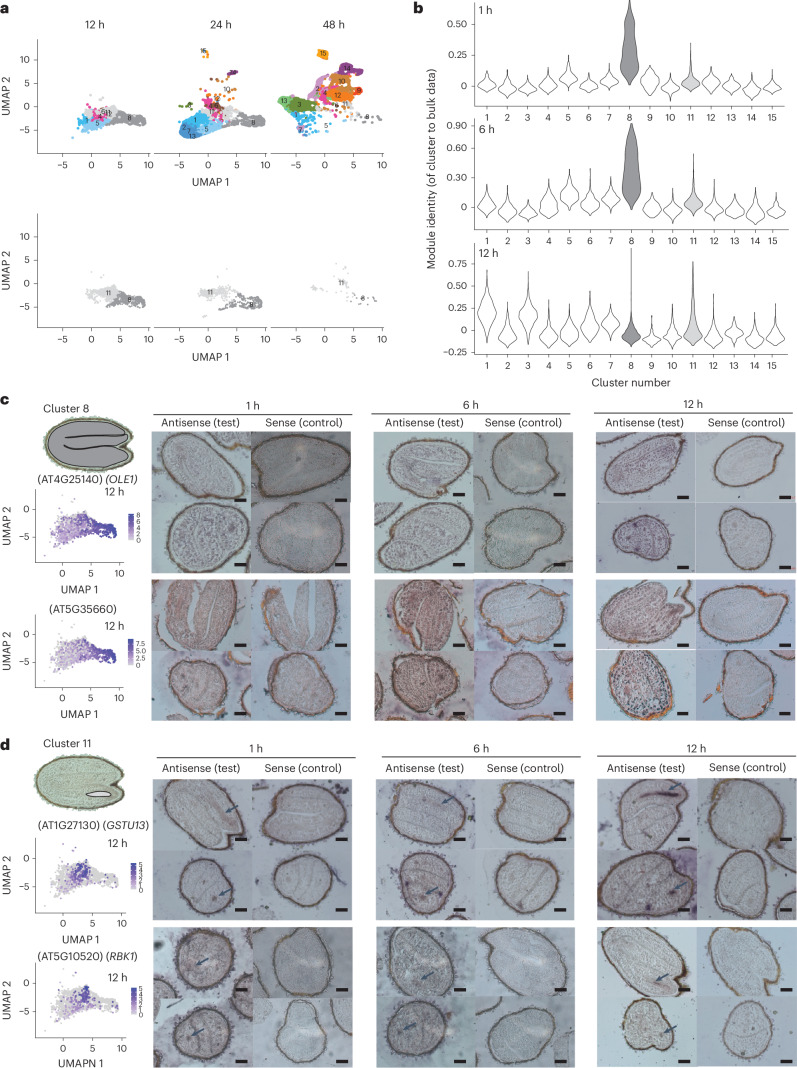
Initial transcriptional stages are established early in germination. **a**, Top, UMAP dimensional reduction and visualization of all cells across three time points (that is, 12, 24 and 48 h), showing the temporal changes in cell and cluster detection. Bottom, UMAP dimensional reduction and visualization of cluster 8 and 11 cells across three time points. **b**, Comparison of transcriptomes of each cell, grouped by cluster, against whole (bulk) seed transcriptomes from early time points during germination. The transcriptomes of cluster 8 cells are strongly similar to the transcriptomes of bulk seeds at 1 and 6 h of germination. **c**,**d**, RNA in situ hybridization to confirm the locations of clusters 8 (**c**) and 11 (**d**) at 1, 6 and 12 h after 48 h of stratification using two cluster-specific marker transcripts for each. Illustrated sketches indicate the area of expression. The expression of each marker is shown in an adjacent UMAP dimensional reduction plot that displays all of the cells detected at 12 h, some of which belong to other clusters. Representative images are shown (more than ten embryos were examined). Scale bars, 200 μm. The arrows indicate regions where signals were detected. Source data

Next, we sought to understand how initial cell transcriptional states are established as cells commence activity. The earliest germination time point, 12 h, was dominated by cells of clusters 8 and 11 (26.74 and 37.27% of cells captured at 12 h, respectively; Fig. 5 and Extended Data Fig. 1f). Like many other clusters, the presence of clusters 8 and 11 was dynamic across germination, being greatest at 12 h, and with the clusters being almost entirely absent by 48 h (Fig. 5a). We were unable to identify known cell type marker transcripts from the published literature with which clusters 8 and 11 could be annotated. Cluster 8 expressed marker transcripts of mature embryos and dry seeds, suggesting that it might comprise cells in an early germination state that have not previously been characterized (Fig. 2c)^41^. To test this idea, we assessed the similarity of the cluster 8 and 11 transcriptomes with the transcriptomes of whole seeds at earlier germination time points (Fig. 5b). To do so, we used a dataset of whole (bulk) seed RNA-seq that included 1, 6 and 12 h germination time points (that is, earlier than in our scRNA-seq analysis) and calculated identity (module) scores between each cell and the bulk seed transcriptomes^13^. Cluster 8 cells identified strongly (more than all other clusters) with the transcriptomes of 1 and 6 h bulk seeds, whereas cluster 11 cells did not. This suggested that the biological properties of clusters 8 and 11 are distinct.

The physical locations of cells in clusters 8 and 11 were determined to better understand their biological properties. We examined the localization of two marker transcripts for each cluster using RNA in situ hybridization. Expression of cluster 8 marker transcripts (AT4G25140 and AT5G35660) was detected throughout the whole embryo (cotyledon, hypocotyl, radicle and provasculature) at 12 h (Fig. 5c, Supplementary Tables 5 and 9 and Extended Data Fig. 5). We also examined the expression domain of cluster 8 marker transcripts at 1 and 6 h, given the similarity of this cluster to earlier bulk germination time points, embryos and dry seeds^41^. The same whole-embryo expression pattern was detected at 1 and 6 h (Fig. 5c and Extended Data Fig. 5). Contrastingly, marker transcripts of cluster 11 (AT1G27130 and AT5G10520) were detected only in the provasculature cells at 1, 6 and 12 h (Fig. 5d, Supplementary Tables 5 and 9 and Extended Data Fig. 5). This expression domain of cluster 11 marker transcripts corresponds to a defined region of abscisic acid and gibberellic acid signalling, which is proposed to regulate the decision to germinate in dormant seeds—an event that precedes the germination events covered by our experiments^4^. Considered together, these data indicate that cluster 8 represents a general cell transcriptional state through which most cells of the embryo pass early in germination. In contrast, cluster 11 probably represents the set of cells where the decision to germinate was made, which appear to have an early germination transcriptional state that is different from all of the other cells.

### Individual gene regulatory programs are active in each cell type

**Fig. 6:**
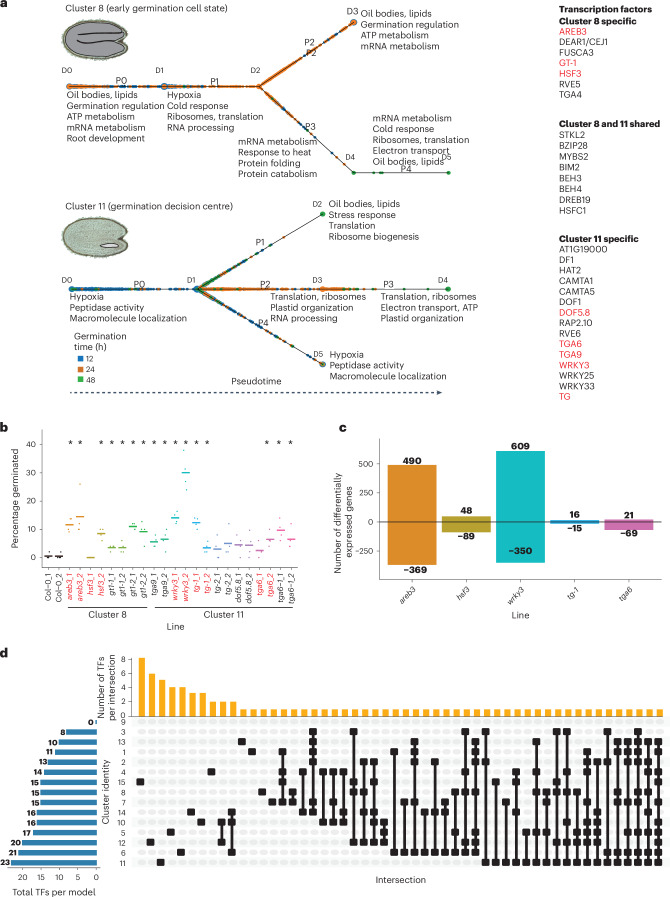
Transcription factors of clusters 8 and 11 are predicted and validated to affect seed germination. **a**, Pseudotime models of cell developmental trajectories for clusters 8 and 11, constructed using CSHMM-TF. P indicates paths and D indicates nodes. Nodes demarcate the start and end of each path. Mutants with mutation of the genes encoding the transcription factors highlighted in red were selected for germination assay. **b**, Germination assay of T-DNA mutant lines predicted in CSHMM-TF of clusters 8 and 11. The percentage of germination of seeds (endosperm rupture) at 24 h is shown. Each data point represents one biological replicate of 50 scored seeds. Seeds from two individual parent plants (_1 and _2) of each mutant line were included. Four biological replicates are included for each mutant line. Asterisks denote significant differences compared with wild-type Col-0. Statistical significance was determined by two-tailed paired Student’s *t*-test (*P* < 1 × 10^−5^). Mutants with mutation of the genes highlighted in red were selected for RNA-seq. **c**, Number of DEGs (FDR < 0.05) in mutants at 24 h compared with wild-type Col-0. Positive values represent upregulation in the mutant and negative values represent downregulation in the mutant. **d**, Active transcription factors (TFs) in every CSHMM-TF model of the 15 clusters identified, comprising a total of 81 unique transcription factors, 39 of which are active in one cell cluster only. Source data

**Extended Data Fig. 6:**
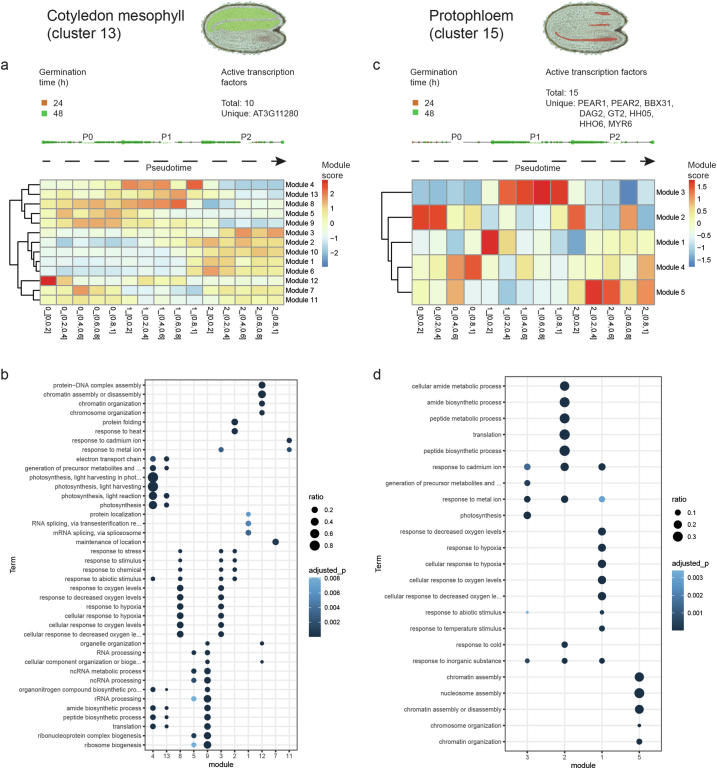
Each cell cluster is defined by a unique gene regulatory program, which reflects dynamic and differing function. **a**, Co-expressed gene modules across the pseudotime trajectory of cluster 13 cotyledon mesophyll cells. **b**, Enriched gene ontology terms of cluster 13. **c**, Co-expressed gene modules across the pseudotime trajectory of cluster 15 protophloem cells. **d**, Enriched gene ontology terms of cluster 15. Gene ontology enrichment p1552 values were determined with one-sided Fisher’s exact test, 1553 adjusted for multiple testing with a Bonferroni correction. Source data

**Extended Data Fig. 7:**
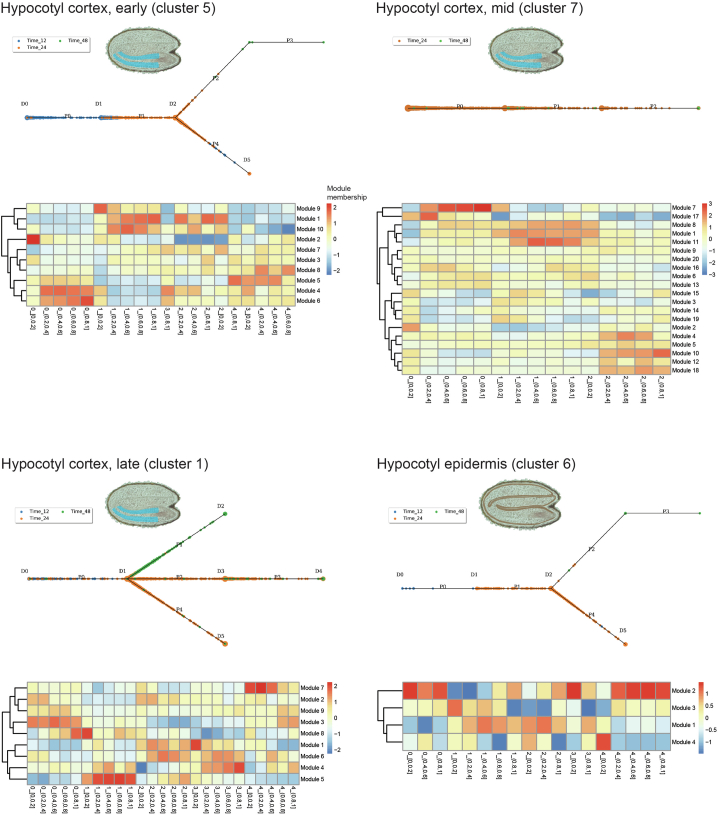
CSHMM-TF models for clusters 5, 7, 1, and 6. CSHMM-TF models for clusters 5 (hypocotyl cortex, early), 7 (hypocotyl cortex, mid), 1 (hypocotyl cortex, late) and 6 (hypocotyl epidermis).

**Extended Data Fig. 8:**
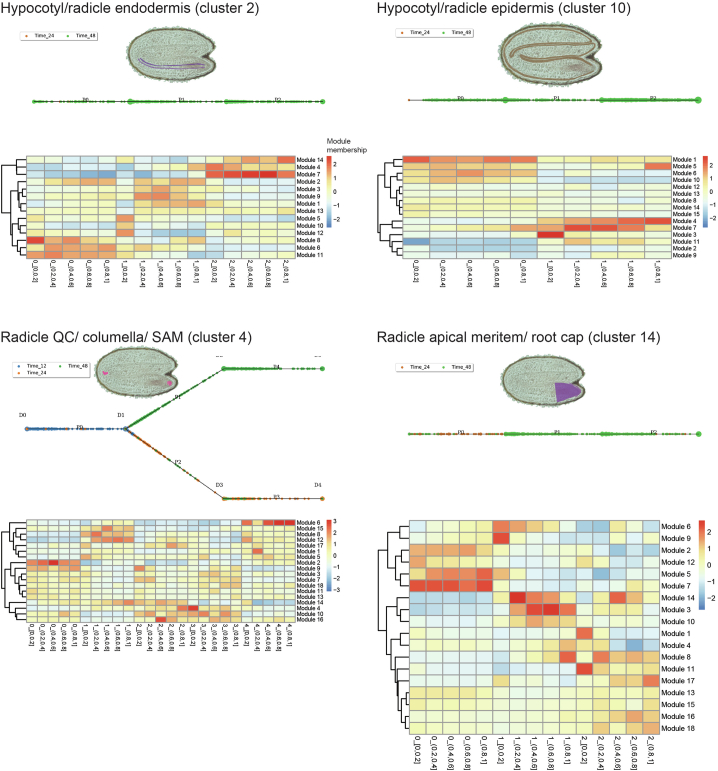
CSHMM-TF models for clusters 2, 4, 10, 14. CSHMM-TF models for clusters 2 (hypocotyl/radicle endodermis), 4 (radicle quiescent centre - QC, shoot apical meristem - SAM, columella), 10 (hypocotyl/radicle epidermis, and 14 (radicle apical meristem region).

**Extended Data Fig. 9:**
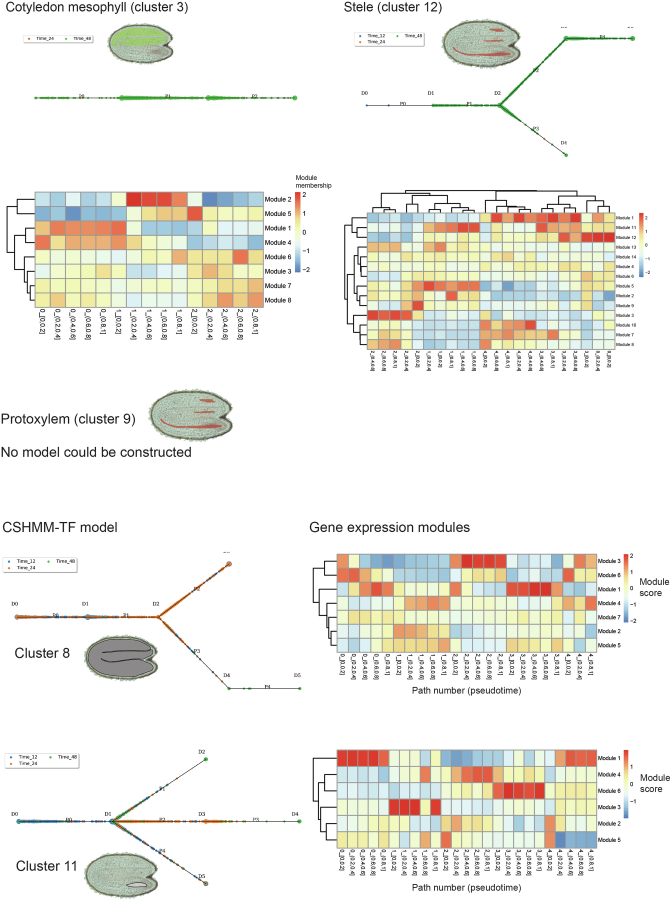
CSHMM-TF models for clusters 3, 12, 9, 8, and 11. CSHMM-TF models for clusters 3 (cotyledon mesophyll), 12 (stele), 9 (protoxylem), 8 and 11.

The many cell types of an embryo each have different roles and contribute at different times to the success of germination^1,4,5,27^. This is achieved by cell types having distinct functional properties, which must be determined by the particular complement of genes that these cells express. Consequently, each cell type of the germinating embryo should have a unique and dynamic gene regulatory program. We examined the properties of these gene regulatory programs by analysing gene expression dynamics and predicting the transcription factors that putatively regulate programs for each cell cluster (that is, cell type or state) identified in our scRNA-seq dataset (Fig. 6, Extended Data Figs. 6–9 and Supplementary Tables 10–14). We used a method called continuous state hidden Markov models—transcription factor (CSHMM-TF) because it assembles pseudotime trajectories from which expression dynamics can be analysed and also predicts which transcription factors are putative regulators of the observed gene expression dynamics^42^. CSHMM-TF predicts regulatory transcription factors based on both their expression and the expression of their target genes. This is achieved by integrating transcription factor binding data, here provided as experimentally validated target genes for >500 *Arabidopsis* transcription factors from genome-wide in vitro protein–DNA binding assays^43^. CSHMM-TF also identifies where the gene expression of groups of cells diverges substantially during pseudotime, splitting cells with different expression states onto different paths that might indicate subpopulations of cells within a cluster.

Gene expression was dynamic and complex across germination in all cell clusters. Each cluster exhibited distinct patterns of gene co-expression across germination and the functional annotations enriched among expressed genes changed across germination, suggesting changes in cell biological properties (Fig. 6a, Extended Data Fig. 5 and Supplementary Tables 10 and 11). For example, cells of cluster 8 (early germination cell state) first expressed genes involved in the utilization of energy resources (ATP and oil bodies; path P0), followed by RNA processing, translation and hypoxia (path P1). The expression of energy biology functions earliest probably indicates the initiation of metabolism, whose resumption when mitochondrial function is being re-established would result in the observed hypoxia signature at this phase of germination^44^. Consistent with this, rice and barley grains express similar functions in the earliest phase of germination^45,46^. Cells then split along two gene expression paths, with path P2 cells expressing genes involved in mRNA metabolism and energy biology, but path P3/P4 cells expressed more protein processing and translation functions. The patterns of gene expression differed in cluster 11 cells (germination decision centre) compared with cluster 8 (Fig. 6a and Supplementary Tables 10 and 11). For example, genes associated with hypoxia were already expressed at the earliest phase of the cluster 11 model (P0). This would be consistent with metabolism in cluster 11 cells becoming active earlier, as would be expected for the cells where the decision to germinate is first made. Cluster 11 cells then split along three gene expression paths (P1, P2/3 and P4), with the expression of functions involved in translation and ribosome biogenesis featured in two of these (P1 and P2/3). For clusters 8 and 11, the pseudotime arrangement was consistent with the actual germination time of cells (Fig. 6a). Similarly complex and distinct patterns of gene expression over germination were observed for all clusters, indicating that gene regulatory programs differ between each cluster and therefore between each cell type or state (Fig. 6, Extended Data Figs. 6–9 and Supplementary Tables 10–14).

Differing gene regulatory programs require the activity of different transcription factors. We first compared the candidate regulatory transcription factors predicted by our models for the gene regulatory programs of clusters 8 and 11. Seven out of 15 predicted regulatory transcription factors were unique to cluster 8, whereas 15 out of 23 were unique to cluster 11 and eight were shared between the two clusters (Fig. 6a). Several of these transcription factors had previous evidence implicating them in germination or early seedling growth. Regulators of brassinosteroid hormone responses were among the transcription factors shared between clusters (BES1-INTERACTING MYC-LIKE PROTEIN 2 (BIM2; AT1G69010), BES1/BZR1 HOMOLOG 3 (BEH3; AT4G18890) and BES1/BZR1 HOMOLOG 4 (BEH4; AT1G78700)), which is notable because brassinosteroids play an important role in cell division and growth^47^. Three transcription factors specific to cluster 8 have functions in embryo development, seed maturation and lipid accumulation (ABA-RESPONSIVE ELEMENT BINDING PROTEIN 3 (AREB3; AT3G56850), FUSCA3 (AT3G26790) and HEAT SHOCK FACTOR 3 (HSF3; AT5G16820))^47–50^. Another cluster 8 specific transcription factor (GT-1; AT1G13450) promotes light-responsive gene expression, consistent with the recent exposure of the seeds to light as a germination trigger^51^. The cluster 11-specific transcription factor ERF55/translucent green (TG; AT1G36060) suppresses light-induced seed germination^52^. Others have roles in mucilage production and root hair growth (DF1; AT1G76880), which occurs early in germination, as well as calcium signalling (CALMODULIN-BINDING TRANSCRIPTION ACTIVATOR 1 (CAMTA1; AT5G09410) and CAMTA5 (AT4G16150)) and auxin-mediated morphogenesis (HAT2; AT5G47370), both of which are signalling pathways that influence seed development and germination^53–59^. These analyses indicate that cells of clusters 8 and 11 execute distinct gene regulatory programs involving different sets of transcription factors.

**Extended Data Fig. 10:**
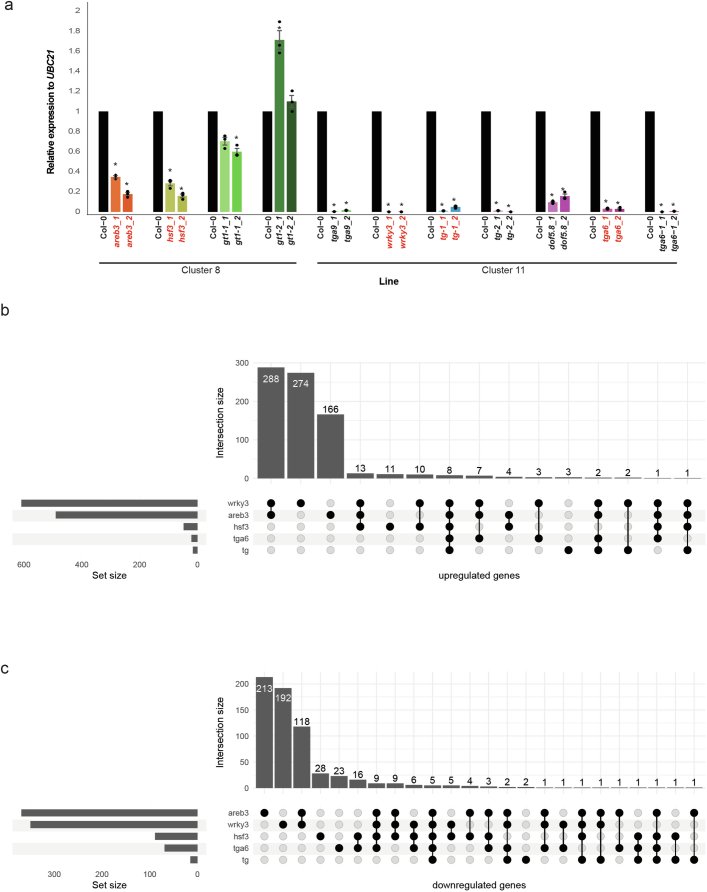
The qRT-PCR and bulk RNA-seq of T-DNA mutant lines. **a**, Validation of target gene expression in t-DNA mutants by qRT-PCR. Three biological replicates are included and data are presented as mean value +/- SEM. Asterisks (*) indicate statistical significance using two-tailed paired Student’s t-test (P < 0.001), **b**,**c**, Overlap of differentially expressed genes (upregulated (**b**) and downregulated (**c**)) between t-DNA mutants. Source data

We assessed the utility of our model as a tool with which to predict transcription factors that influence germination phenotypes. Our analysis focused on the predicted regulatory transcription factors specific to cluster 8 (7 out of 15) and cluster 11 (15 out of 23). We obtained transfer DNA (T-DNA) fragments conferring homozygous mutations expected to disrupt either promoters or coding sequences of the transcription factors from cluster 8 (four independent T-DNA fragments disrupting three genes: *areb3* for *AREB3*, *gt1-1* and *gt1-2* for *GT1*, and *hsf3* for HSF3) and cluster 11 (seven independent T-DNA fragments disrupting five genes: *dof5.8* for AT5G66940, *tg-1* and *tg-2* for *TG*, *tga6* and *tga6-1* for *TGA6* (AT3G12250), *tga9* for *TGA9* (AT1G08320) and *wrky3* for AT2G03340; Supplementary Table 15). These genes were selected for analysis based on the availability of single-insertion mutation-causing lines from the Arabidopsis Biological Resource Center targeting the intended region of genes. Progeny from two parent lines were collected for each T-DNA fragment for quantitative PCR with reverse transcription (qRT-PCR) analysis to confirm knockout or knockdown of the target genes. The results indicated that fragments targeting *AREB3*, *HSF3*, *GT1* and *DOF5.8* caused knockdown of the genes, whereas fragments targeting *TGA9*, *WRKY3*, *TG* and *TGA6* caused knockout of the genes. The second allele for *GT1*, for which the insertion of T-DNA was in the promoter region, caused overexpression of this gene in the mutant (Extended Data Fig. 10a). The germination rates of the mutated plants (as determined by testa rupture) were compared with those of wild-type Col-0 after 24 h exposure to light, across four independent biological replicates (Fig. 6b). The germination rates were faster for all mutants with mutations affecting four out of the eight transcription factors (AREB3, GT1, TGA9 and WRKY3). For cluster 8, the germination rates were consistently faster in all progeny lines of *areb3*, *gt1-1* and *gt1-2* mutants. The germination rates of the *hsf3* progeny lines were inconsistent. For cluster 11, the germination rates were consistently faster in all progeny lines of *tga9* and *wrky3*, whereas the germination rate of *dof5.8* did not differ from that of the wild type. *tg-1* exhibited an increased germination rate but *tg-2* did not, suggesting a difference of effect between these two mutations in the same gene. The germination rate was increased in one of two *tga6* progeny lines and both *tga6-1* progeny lines, potentially resulting from a relatively weak phenotypic effect of *tga6*. Overall, these results indicate that our modelling approach is able to identify transcription factors that influence germination phenotypes when altered through mutation of their encoding genes.

We also investigated the effects of mutating genes encoding predicted regulatory transcription factors on global gene expression. A subset of mutants were selected to represent a spectrum of phenotypes: *areb3* and *wrky3* with strong effects on germination rate; *tg-1* with a moderate effect; and *hsf3* and *tga6* with weak or inconsistent effects (Fig. 6b; selected lines are indicated in red). The transcriptomes of these mutants were compared with those of wild-type plants at 24 h of germination using bulk RNA-seq (Fig. 6c and Supplementary Table 16). Expression values of the target mutated genes were consistent with qRT-PCR results for each line. The mutants *areb3* and *wrky3*, which had the strongest increases in germination rates, also had the largest numbers of differentially expressed genes (DEGs) (859 for *areb3* and 959 for *wrky3*), whereas only 31 genes were misregulated in *tg-1*. There was a large and significant overlap in DEGs between mutants (Extended Data Fig. 10b,c; *P* < 1 × 10^−5^; hypergeometric test), which is consistent with both similar gene networks being affected and also the common effect of accelerated germination. The downregulated genes in the *areb3* mutant were significantly enriched for targets of the AREB3 transcription factor (27 out of the 369 DEGs from 536 total known AREB3 targets; *P* < 1 × 10^−5^; hypergeometric test), but the DEGs of the other mutants were not significantly enriched for the known targets of those transcription factors^43^. Overall, these differential expression results combined with the early germination phenotypes indicate that AREB3 and WRKY3 may influence gene expression during germination, further supporting the utility of our modelling approach. The bulk RNA-seq approach used here lacks sensitivity given that we expect these transcription factors to operate within defined cell subsets and at specific times, so the results suggest that analysis of mutants at the single-cell level may be able to resolve the cell state-specific roles of these transcription factors in germination.

Having validated the utility of our modelling approach to identify candidate transcription factors that may influence germination phenotypes, we examined the characteristics of the gene regulatory program models of all cell clusters (Fig. 6d, Extended Data Figs. 6–9 and Supplementary Tables 10–14). A total of 81 transcription factors were predicted to be active across the models of all cell clusters (Fig. 6d and Supplementary Table 17). Mutations in genes encoding 15 of the 81 transcription factors led to independently reported phenotype changes relevant to seed development, germination and early seedling growth (Supplementary Table 17). These include the previously discussed mutations of *AREB3* and *TG*. Mutations of *WRKY DNA-BINDING PROTEIN 18* (*WRKY18*; *AT4G31800*) confer altered sensitivity to abscisic acid and hence an altered germination rate^60^. Mutation of *DOF AFFECTING GERMINATION 2* (*DAG2*; AT2G46590) results in a decreased germination rate in the light, suggesting that DAG2 is a positive regulator of light-mediated seed germination with expression confined to vascular tissue and induced by imbibition^61,62^. *MYB HYPOCOTYL ELONGATION-RELATED* (*MYBH*; AT5G47390) overexpression caused increased hypocotyl elongation of seedlings grown in the light^63^. Thirty-nine of the 81 transcription factors were unique to a single cluster, such as PHLOEM EARLY DOF 1 (PEAR1; AT2G37590) and PHLOEM EARLY DOF 2 (PEAR2; AT5G02460), which were uniquely predicted to regulate gene expression in the protophloem (cluster 15) and which are known regulators of protophloem development^64^ (Supplementary Table 17). In contrast, other transcription factors were shared between many cell types, such as BIM2 (a regulator of brassinosteroid signalling and growth; eight clusters) and MYBS2 (AT5G08520; a known regulator of glucose and abscisic acid signalling; nine clusters)^47,65^.

Overall, these analyses indicate that the different cell types of a germinating embryo express unique and dynamic gene regulatory programs that are probably governed by specific sets of transcription factors, and support the ability of our models to identify relevant candidate transcription factors involved in germination that may be used as a resource in other studies.

## Discussion

How cell type-specific patterns of gene expression are established and change in individual embryo cells during germination are notable unanswered questions in seed biology. In this study, we have comprehensively described gene expression dynamics between 12 and 48 h of germination in the individual cells of the *Arabidopsis* embryo. We observed that gene expression is highly dynamic within individual cell types and that cells transition through distinct transcriptional states as germination progresses. The earliest specific transcriptional activity is detected in the root tip vasculature, supporting the definition of this region as the germination decision centre^4^. Aside from this, nearly all other embryo cells pass through a common transcriptional state at the start of germination. This was evident from our observations that more than one-third of cells at 12 h of germination belonged to a single cluster (cluster 8) and that marker transcripts for this cluster were broadly expressed across the embryo when observed using RNA in situ hybridization. We could rule out that the decreased transcriptome complexity observed at 12 h was due to undersampling as the cells at this time point had the highest number of reads per cell and the highest sequencing saturation. Additionally, cells with similarly low UMI counts from later time points clustered away from the early germination clusters. This confirmed that the RNA content of cells at 12 h is genuinely low and more homogeneous than at later time points.

Subsequently, the many cell type-specific gene expression programs of the embryo emerge. Why cells need to express this shared transcriptional program upon first activity remains to be discovered, especially given that their cell identity has already been defined during embryogenesis. The emergence of specialized transcriptional states from a common ground state also poses the question of which factors provide the memory of cell identity after embryo development and seed dessication. These may include factors such as specific mRNAs stored at low abundance, stored transcription factors or preserved epigenetic differences between cell types.

The transcriptomes of each embryo cell type changed substantially as germination progressed, resulting in changes to the molecular pathways and functions expressed by each cell type over time. The hypocotyl cortex cells were an example of this, expressing genes involved in mRNA splicing and transcriptional functions early in germination, progressing to protein maturation and the establishment of photosynthesis in mid-germination and then chloroplast organization and cell growth in late germination when the seed–seedling transition occurs. Similar dynamics were observed in every cell type, but in each case the functions expressed and the sequence of changes were specific to the individual cell type. This presumably reflects the unique role of each embryo cell type during germination. Underlying these expression dynamics were cell type-specific gene regulatory networks, defined by groups of transcription factors. Although some transcription factors were predicted to be active across multiple cell types, a subset of transcription factors were specific to individual cell types or transcriptional states. This indicates that distinct groups of transcription factors govern the dynamic functional changes of each embryo cell type as germination progresses. Mutations of a subset of transcription factors from two clusters (cluster 8 (common transcriptional state) and cluster 11 (germination decision centre)) resulted in faster germination. This suggests that genes expressed in clusters 8 and 11 are enriched for germination inhibitors that are active at the earliest stages of germination. While it may initially appear counter-intuitive that genes encoding germination inhibitors are induced during germination, this may be explained by those genes undergoing feedback inhibition. In this case, an increase in transcription of the germination inhibitor genes would result in suppression of their activity. Feedback inhibition is common and well documented among germination genes, including many components of gibberellin synthesis, degradation and response^4,66^. These observations also highlight that transcript abundance alone cannot predict gene function. The transcriptional induction of germination inhibitors at the early stages of the germination program may represent a regulatory mechanism that merits further examination.

Overall, we illustrate that the cells of the embryo progress through specific transcriptional states as germination progresses. This enables individual cell types to express the genes that define the changing functions of those cell types at the appropriate time, thereby contributing to the successful seed–seedling transition. Seed structures and resources vary remarkably between plant species, requiring different cell types, functions and dynamics. Our study provides a framework for the analysis of functional variation in seeds between species and for investigation of how different species establish cell transcriptional states in the embryo. The interactive version of the atlas from this study, available at https://scgerminationatlas.latrobe.edu.au/, can be mined as a resource for future detailed studies of germination and cell types.

## Methods

### Plant material and growth conditions

Col-0 and Cvi-0 seeds were sown on MS media plates (containing 3% sucrose). Seeds were sterilized and subjected to 48 h of cold (4° C), dark stratification before being transferred to continuous light (at 22° C), then collected after 12, 24 or 48 h in the light. Two biological replicates were collected and analysed.

### Germination assay

The germination of Col-0 was scored by measuring both testa rupture and endosperm rupture after 12, 24, 36 and 48 h in the light. Four biological replicates consisting of 50 seeds were included for each time point for the measurement. Testa rupture was scored when visible slits formed on the surface of the seed coat, whereas endosperm rupture was scored after the radicle tip emerged out of the seed coat^1^.

### Dissociation of *Arabidopsis* embryos into single cells

Approximately 1,000 seeds per individual sample were sandwiched between two glass slides and embryos were released mechanically from seed coats by pressing the slides together. Ruptured seed coats and embryos were collected into microcentrifuge tubes and separated from each other using a Percoll gradient. In brief, the samples were resuspended in MC buffer (10 mM potassium phosphate (pH 7.0), 50 mM NaCl and 0.1 M sucrose) and loaded onto a 1 ml 60% Percoll cushion. The tubes were then centrifuged at 800*g* for 5 min and the embryo fraction (at the bottom of the tubes) was collected and resuspended in 0.6 ml MC buffer. A second Percoll gradient was repeated to obtain pellets of embryos without any seed coats. Enriched embryos were cut with razor blades and digested in protoplasting buffer (2% wt/vol cellulase, 3% wt/vol macerozyme, 0.4 M d-mannitol, 20 mM MES, 20 mM KCl in Milli-Q water (with the pH adjusted to 5.7 using 1 M Tris/HCL (pH 7.5)), 0.1% wt/vol bovine serum albumin, 10 mM CaCl_2_ and 5 mM β-mercaptoethanol). After 3 h of digestion, protoplasts were filtered through a 70 μm cell strainer, followed by a 40 μm cell strainer to remove debris, and centrifuged at 500*g* for 5 min. The supernatant was removed and the pellet was washed with 2 ml protoplasting buffer without enzymes or mercaptoethanol and centrifuged at 500*g* for 5 min. The pellet was resuspended in 50 μl protoplasting buffer without enzymes or mercaptoethanol. Protoplasts were counted using a haemocytometer and adjusted to a final concentration of 800–1,200 protoplasts per μl.

### Bulk RNA-seq library preparation

Col-0 seeds were sown and collected at the 12, 24 and 48 h time points as above in three independent replicates (batches) of the experiment. Embryos were released from seed coats and enriched by performing a Percoll gradient. For bulk RNA-seq, embryos were collected either without protoplasting or with protoplasting. RNA was extracted using a Qiagen RNeasy Plant Mini Kit. RNA quality and integrity were determined using a Qubit fluorometer. Libraries were prepared using a TruSeq Stranded mRNA Library Prep Kit and sequenced with an Illumina NextSeq 500 sequencer using 75 bp single-end kits.

### Bulk RNA-seq analysis

Raw Illumina reads were trimmed for quality and adaptor sequences with TrimGalore version 0.4.5. Trimmed fastq files were quality checked with FastQC^67^ and aligned to the *Arabidopsis thaliana* Col-0 TAIR10 assembly with HISAT2 version 2.1.0 (ref. ^68^). Exonic counts aggregated by genes were calculated with FeatureCounts from the Rsubread package (version 1.6.2)^69^ using the Araport11 annotation^70^. Differential expression analysis between the dissociated and non-dissociated embryos was performed in R 3.5.1 (ref. ^71^) with the edgeR package^72,73^. The design matrix was defined as model.matrix (time + protoplast) and generalized linear models for each gene were fit with glmFit. Genes differentially expressed by the dissociation treatment were identified by performing a likelihood ratio test on the protoplast factor with glmLRT. We imposed a 1% FDR and a minimum absolute log_2_[fold change] of 1.5 to call genes as significantly induced or repressed by the dissociation. Similarly, to evaluate the coherence of the transcriptional dynamics of whole embryos versus isolated protoplasts through germination, we identified developmentally regulated genes by performing a likelihood ratio test on the time factor with glmLRT for whole and dissociated embryos separately. We imposed a 5% FDR and a minimum absolute log_2_[fold change] of 1.5 successive time points to call genes as significantly induced or repressed in that time interval. We then quantified the number of genes with discordant changes between whole and dissociated embryos.

### Single-cell RNA-seq library preparation

Six thousand protoplasts per time point and replicate were loaded onto a Chromium Single Cell instrument (10X Genomics, Millennium Science) to generate single-cell Gel bead-in-EMulsions (GEMs). scRNA-seq libraries were generated with the Chromium Single Cell 3′ Library & Gel Bead Kit v2 (10X Genomics, Millennium Science) according to the user guide (Chromium Single Cell 3′ Reagent Kits v2). The resulting libraries were checked on an Agilent 2100 Bioanalyzer and quantified with the KAPA Library Quantification Kit for Illumina Platforms (Millennium Science). The libraries were sequenced on an Illumina NextSeq 500 using two 150 bp paired-end kits with 15% PhiX. The raw scRNA-seq dataset comprised 26 bp read 1, 116 bp read 2 and 8 bp i7 index reads.

### Single-cell RNA-seq analysis

CellRanger count (version 1.3.0) was used to generate raw UMI count matrices for each sample separately, mapping to the TAIR10 *A. thaliana* genome masked for Cvi-0 SNPs with the STAR options --alignIntronMin = 10 --alignIntronMax = 5000 --scoreDelOpen = −1 --scoreDelBase = −1 --scoreInsOpen = −1 --scoreInsBase = −1 and using the Araport11 AtRTD2 gene transfer format (GTF) file.

Single cells for the first replicate, containing Cvi-0 and Col-0 cells, were genotyped by first counting the UMIs containing Col-0 or Cvi-0 SNPs for each cell barcode, followed by density-based clustering with DBSCAN. These steps are included in the sctools package (https://github.com/timoast/sctools; version 0.7). The clustering parameters were optimized for each sample: *ϵ*_background_ = 0.5 and *ϵ*_margin_ = 0.3 for 12 h replicate 1; *ϵ*_background_ = 0.4 and *ϵ*_margin_ = 0.3 for 24 h replicate 1; and *ϵ*_background_ = 0.4 and *ϵ*_margin_ = 0.15 for 48 h replicate 1. Only cells genotyped as Col-0 were retained for subsequent biological analysis.

We applied emptyDrops^74^ from the DropletUtils package (version 1.6.1) following the guide to distinguish real cells. Further quality control was performed using scater (version 1.14.6)^75^ to remove cells with: (1) more than three median absolute deviations of the log_10_ read counts below the median values; and (2) more than three median absolute deviations of the log_10_ genes detected below the median. Then, calculateAverage was used to remove low-abundance genes with an average count below 0.

Pseudobulk expression by time point was calculated with Seurat’s AggregateExpression function. The correlation of normalized log[counts per million] between pseudobulk and non-protoplasted bulk controls was calculated with the Pearson method, with and without removing protoplast-responsive genes.

Genes induced during protoplast isolation were removed before applying the normalization method calculateSumFactors with pool-based size factors used from scran (version 1.10.2)^76^. Highly variable genes were selected by modelGeneVar and getTopHVGs with the biological variance threshold set to 0. FastMNN^77^ was then performed using highly variable genes to integrate datasets from each sample. Mutual nearest neighbours dimension reductions were applied to construct a shared nearest neighbour graph with the function provided in scran, and the Louvain algorithm from igraph (version 1.2.2)^78^ was followed to group cells into clusters. Mutual nearest neighbours dimension reductions were also applied to generate a two-dimensional uniform manifold approximation and projection for visualization.

### Cluster and cell type annotation

To identify cluster-enriched genes, genes upregulated in each cluster were calculated using FindMarkers from Seurat (4.0.5) with the *P* value < 0.01 (ref. ^79^) (Supplementary Table 2). The cell type of each cluster was manually annotated and assigned using known cell type marker genes from the literature (Supplementary Table 3). Well-characterized endosperm and seed coat marker genes were included to show exclusion of these two tissues and enrichment of embryos in the current data.

### Comparison with bulk RNA-seq data

We also compared the scRNA-seq data with the published time series sequencing bulk RNA-seq profiles of seed germination^13^. The samples used for bulk RNA-seq were collected after 48 h of dark stratification, followed by 1, 6, 12 and 24 h of exposure to continuous light. Differential expression analysis between samples from a specific time point to others was performed with the limma package (version 3.38.3)^80^. With the design matrix defined as model.matrix (0 + time), precision weights were calculated by voom^81^ and used in eBayes for statistical testing. Contrasts were made between data from every time point to the average of data from other time points. Genes that were differentially expressed with the log_2_[fold change] above 1.5 and FDR < 5% were retained as differentially expressed genes. We then filtered out genes that were regarded as differentially expressed genes at more than one time point. Then, the lists of unique differentially expressed genes of individual time points were used in the scRNA-seq data to calculate their average expression in each of the cells with the function AddModuleScore^82^.

### RNA in situ hybridization

Seeds were harvested and fixed in ice-cold Farmer’s fixative (3:1 ethanol:acetic acid). The samples were placed in a cold room (4° C) overnight. The following day, the fixed tissues were dehydrated using a Leica TP1020 Semi-Enclosed Benchtop Tissue Processor (Leica Biosystems) at room temperature in a graded series of ethanol (1 h each at 75, 85, 100, 100 and 100% vol/vol). The tissues were then transferred to a graduated ethanol:xylene series (1 h 20 min each at 75%:25%, 50%:50% and 25%:75% vol/vol), finished with a xylene series (1 h each at 100 and 100% vol/vol). Tissue was then added to molten Surgipath Paraplast Paraffin (Leica Biosystems) twice for 2 h each at 65° C. Paraplast blocks were then prepared with dozens of seeds in each block using the Leica EG1150 H Heated Paraffin Embedding Module with the added Leica EG1150 C Cold Plate for Modular Tissue Embedding System (Leica Biosystems) with vacuum infiltration. Embedded tissues were cut at 8 µm sections and in situ hybridization was carried out according to a modified protocol from Jackson^83^: 50° C hybridization temperature and 0.2× saline-sodium citrate (SSC) washes. Transcripts of interest were amplified using designed primers (Supplementary Table 5) and cloned into pGEM-T Easy vector (Promega). Digoxigenin-labelled antisense and sense RNA probes were transcribed from the T7 or SP6 promoter of pGEM-T Easy vector (Promega) according to the manufacturer’s instructions. All hybridization results were observed and photographed using a Zeiss Axio Observer A1 microscope (Carl Zeiss). Representative images are shown with more than ten embryos examined.

### Trajectory inference analysis

Monocle 3 (1.0.0)^84^ was used to construct single-cell trajectories. Cells from annotated hypocotyl clusters were extracted and reprocessed (including normalization and batch effect correction^77^, dimensionality reduction and clustering) with Monocle 3. This resulted in three distinct partitions and we learned the trajectory for each of the partitions. We selected the beginning nodes of the trajectory where more adjacent cells come from 12 h. Modules of co-regulated genes were then calculated using Louvain community analysis based on genes with the function find_gene_modules. Aggregated expression of all genes in each module across cells along pseudotime was plotted in a heatmap. After grouping modules based on their expression pattern according to the pseudotime stage, we assessed enriched Gene Ontology terms using all genes from each stage. Gene Ontology analysis was performed using the topGo package (version 2.34.0)^85^.

### Inferring transcription factor activation order with CSHMM-TF

CSHMM-TF (commit 417bd71; running Python 2.7)^42^ was used for analysis of time series single-cell expression data with information about transcription factors (transcription factor binding data from ref. ^43^). Cells from each cluster were extracted separately and their raw count matrix and information about their collected time were used as input. find_gene_modules was used to find gene modules across individual clusters. Aggregated expression was then calculated based on assigned time blocks of activation along the learned path by CSHMM-TF. Gene Ontology analysis was performed in each module of genes separately and the results are shown in dot plots, with size representing the ratio of provided genes to all genes in a specific term and colour representing adjusted enrichment *P* values.

### Gene Ontology analysis

Gene Ontology analysis was performed using the topGO package^85^. Whole gene sets without protoplasting genes were used as the background. Adjusted *P* values of gene enrichment were obtained from multiple *P* values generated from topGO with the number of tests run in each enrichment analysis.

### Characterization and bulk RNA-seq of T-DNA lines

T-DNA lines^86^ were genotyped by PCR with gene-specific primers designed using T-DNA express (http://signal.salk.edu/cgi-bin/tdnaexpress) and T-DNA primers for the different mutant collections^87,88^ (Supplementary Table 15). Target gene expression in T-DNA mutants was validated by qRT-PCR (Supplementary Table 15). The germination rates of homozygous mutant lines were then assessed after 24 h in light. Seeds from two individual parent plants of each mutant line were included. Endosperm rupture was counted in four biological replicates of 50 seeds. Seeds of mutant lines and the Col-0 wild type were then collected at 24 h for RNA extraction using the Spectrum Plant Total RNA Kit (Sigma–Aldrich), followed by bulk RNA-seq library preparation and analysis.

### Reporting summary

Further information on research design is available in the Nature Portfolio Reporting Summary linked to this article.

## Supplementary information

Reporting Summary

Supplementary Tables Supplementary Tables 1–17.

## Source data

Source Data Fig. 1 Protoplast isolation-responsive genes.

Source Data Fig. 2 Cluster-specific markers from the literature.

Source Data Fig. 4 All 157 genes per module.

Source Data Fig. 5 Marker gene list for clusters 8 and 11.

Source Data Fig. 6 Cluster-specific and known transcription factors.

Source Data Extended Data Fig. 3 Marker gene list for clusters 1, 5 and 7

Source Data Extended Data Fig. 4 Marker gene list for combined clusters 1 5 and 7

Source Data Extended Data Fig. 6 All models Gene Ontology summary

Source Data Extended Data Fig. 10 DEGs in T-DNA mutants

## Data Availability

Raw and processed bulk and scRNA-seq data are available from the EBI database ArrayExpress under the accession codes E-MTAB-12521 (bulk protoplasting controls), E-MTAB-13449 (transcription factor mutant RNA-seq) and E-MTAB-12532 (single-cell data). The interactive web browser can be found at https://scgerminationatlas.latrobe.edu.au/. This study used the TAIR10 assembly with the Araport11 AtRTD2 annotation (https://www.araport.org/), the transcription factor binding dataset from ref. ^43^ and the bulk RNA-seq time course dataset from ref. ^13^. Source data are provided with this paper.
