# Supplementary material for: Establishment of single-cell transcriptional states during seed germination
Source: Nat Plants. 2024 Sep 10;10(9):1418–34. doi: 10.1038/s41477-024-01771-3 (PMC11410669; doi:10.1038/s41477-024-01771-3)
Supplement: Supplementary file 1 — Reporting Summary [file 41477_2024_1771_MOESM1_ESM.pdf]

nature portfolio

Corresponding author(s): James Whelan, Quentin Gouil, Mathew G Lewsey

Last updated by author(s): Jun 19, 2024

Reporting Summary

Nature Portfolio wishes to improve the reproducibility of the work that we publish. This form provides structure for consistency and transparency in reporting. For further information on Nature Portfolio policies, see our Editorial Policies and the Editorial Policy Checklist.

Statistics

For all statistical analyses, confirm that the following items are present in the figure legend, table legend, main text, or Methods section.

n/a	Confirmed	
☐	☒	The exact sample size (*n*) for each experimental group/condition, given as a discrete number and unit of measurement
☐	☒	A statement on whether measurements were taken from distinct samples or whether the same sample was measured repeatedly
☐	☒	The statistical test(s) used AND whether they are one- or two-sided<br>*Only common tests should be described solely by name; describe more complex techniques in the Methods section.*
☒	☐	A description of all covariates tested
☐	☒	A description of any assumptions or corrections, such as tests of normality and adjustment for multiple comparisons
☒	☐	A full description of the statistical parameters including central tendency (e.g. means) or other basic estimates (e.g. regression coefficient) AND variation (e.g. standard deviation) or associated estimates of uncertainty (e.g. confidence intervals)
☐	☒	For null hypothesis testing, the test statistic (e.g. *F*, *t*, *r*) with confidence intervals, effect sizes, degrees of freedom and *P* value noted<br>*Give P values as exact values whenever suitable.*
☒	☐	For Bayesian analysis, information on the choice of priors and Markov chain Monte Carlo settings
☒	☐	For hierarchical and complex designs, identification of the appropriate level for tests and full reporting of outcomes
☐	☒	Estimates of effect sizes (e.g. Cohen's *d*, Pearson's *r*), indicating how they were calculated

Our web collection on statistics for biologists contains articles on many of the points above.

Software and code

Policy information about availability of computer code

Data collection	10X Genomics CellRanger v1.3.0 was used.
Data analysis	We used R v3.5.1, Trim Galore! vO.4.5, Hisat2 v2.1.0, edgeR V3.24.3, DropletUtils (vl.6.1), scater vl.14.6, scran vl.10.2, igraph vl.2.2, Seurat v4.0.5, limma V3.38.3, topGO v2.34.0, python 2.7, CSHMM-TF (https://github.com/jessical338/CSHMM-for-time-series-scRNA-Seq, commit 417bd71.),Monocle 3 v1.0.0, FeatureCounts v1.6.2, sctools v0.7

For manuscripts utilizing custom algorithms or software that are central to the research but not yet described in published literature, software must be made available to editors and reviewers. We strongly encourage code deposition in a community repository (e.g. GitHub). See the Nature Portfolio guidelines for submitting code & software for further information.

Data

Policy information about availability of data

All manuscripts must include a data availability statement. This statement should provide the following information, where applicable:

- Accession codes, unique identifiers, or web links for publicly available datasets
- A description of any restrictions on data availability
- For clinical datasets or third party data, please ensure that the statement adheres to our policy

Raw and processed bulk and single-cell RNA-seq data are available at EBI Annotare under accessions E-MTAB-12521 and E-MTAB-12532. Bulk RNA-seq for transcription factor mutant is under accession E-MTAB-13449. The interactive web browser can be found at https://scgerminationatlas.latrobe.edu.au. This

study used the TAIR10 assembly with the Araport11 AtRTD2 annotation (https://www.araport.org/), the TF-binding dataset from O' Malley et al. 2016, and the bulk RNA-seq time-course dataset from Narsai et al. 2017

Human research participants

Policy information about studies involving human research participants and Sex and Gender in Research.

Reporting on sex and gender	N/A
Population characteristics	N/A
Recruitment	N/A
Ethics oversight	N/A

Note that full information on the approval of the study protocol must also be provided in the manuscript.

Field-specific reporting

Please select the one below that is the best fit for your research. If you are not sure, read the appropriate sections before making your selection.

☒ Life sciences ☐ Behavioural & social sciences ☐ Ecological, evolutionary & environmental sciences

For a reference copy of the document with all sections, see nature.com/documents/nr-reporting-summary-flat.pdf

Life sciences study design

All studies must disclose on these points even when the disclosure is negative.

Sample size	Sample size calculation did not apply. For each single-cell RNA-seq sample we aimed to load 6,000 cells on the 10X Chromium machine to obtain optimal data quality (maximising number of cells captured while limiting doublet rate). We reasoned that these cell numbers should allow good representation of all the major cell types in an Arabidopsis seed.
Data exclusions	We excluded non-Col-0 cells from the analysis. We initially mixed Cvi-0 and Col-0 cells for single-cell RNA-seq to determine true cells (containing SNPs from only one ecotype) from background or doublets, then used these parameters to analyse only the Col-0 cells.
Replication	We conducted the single-cell time-series in duplicates. We present the integrated data. Both replication attempts were successful. A third replicate, while technically also successful, was excluded after discovering the seeds were not of the expected genotype.
Randomization	Not applicable (no experimental groups)
Blinding	Not applicable (no experimental groups)

Reporting for specific materials, systems and methods

We require information from authors about some types of materials, experimental systems and methods used in many studies. Here, indicate whether each material, system or method listed is relevant to your study. If you are not sure if a list item applies to your research, read the appropriate section before selecting a response.

Materials & experimental systems

n/a	Involved in the study	
☒	☐	Antibodies
☒	☐	Eukaryotic cell lines
☒	☐	Palaeontology and archaeology
☒	☐	Animals and other organisms
☒	☐	Clinical data
☒	☐	Dual use research of concern

Methods

n/a	Involved in the study	
☒	☐	ChIP-seq
☒	☐	Flow cytometry
☒	☐	MRI-based neuroimaging
